# Reducing the Diagnostic Heterogeneity of Schizoaffective Disorder

**DOI:** 10.3389/fpsyt.2017.00018

**Published:** 2017-02-10

**Authors:** Katherine Seldin, Kristan Armstrong, Max L. Schiff, Stephan Heckers

**Affiliations:** ^1^Psychiatric Neuroimaging Program, Department of Psychiatry and Behavioral Sciences, Vanderbilt University, Nashville, TN, USA

**Keywords:** schizoaffective disorder, schizophrenia, outcome, nosology, psychosis, clinical characteristics

## Abstract

**Objective:**

Clinical outcome studies of schizoaffective disorder patients have yielded conflicting results. One reason is the heterogeneity of samples drawn from the schizoaffective disorder population. Here, we studied schizoaffective disorder patients who showed marked functional impairment and continuous signs of illness for at least 6 months (i.e., DSM criteria B and C for schizophrenia).

**Methods:**

We assessed 176 chronic psychosis patients with a structured interview (SCID-IV-TR) and the Diagnostic Interview for Genetic Studies schizoaffective disorder module. We diagnosed 114 patients with schizophrenia and 62 with schizoaffective disorder. The two groups were similar with regard to age, gender, and race. We tested for group differences in antecedent risk factors, clinical features, and functional outcome.

**Results:**

The schizoaffective disorder group differed from the schizophrenia group on two measures only: they showed higher rates of suicidality (more suicide attempts, *p* < 0.01; more hospitalizations to prevent suicide, *p* < 0.01) and higher anxiety disorder comorbidity (*p* < 0.01).

**Conclusion:**

When schizoaffective disorder patients meet DSM criteria B and C for schizophrenia, they resemble schizophrenia patients on several measures used to assess validity. The increased rate of anxiety disorders and suicidality warrants clinical attention. Our data suggest that a more explicit definition of schizoaffective disorder reduces heterogeneity and may increase validity.

## Introduction

A recent meta-analysis of 50 studies comparing illness course and clinical outcomes in schizoaffective disorder, schizophrenia, and affective disorders reports the use of 10 different sets of diagnostic criteria for schizoaffective disorder (1). A review of the schizoaffective disorder literature revealed that patients with schizoaffective disorder had a better outcome than those with schizophrenia when ICD-10 criteria were used, but not when DSM-IV criteria were used (2).

**Figure 1:**
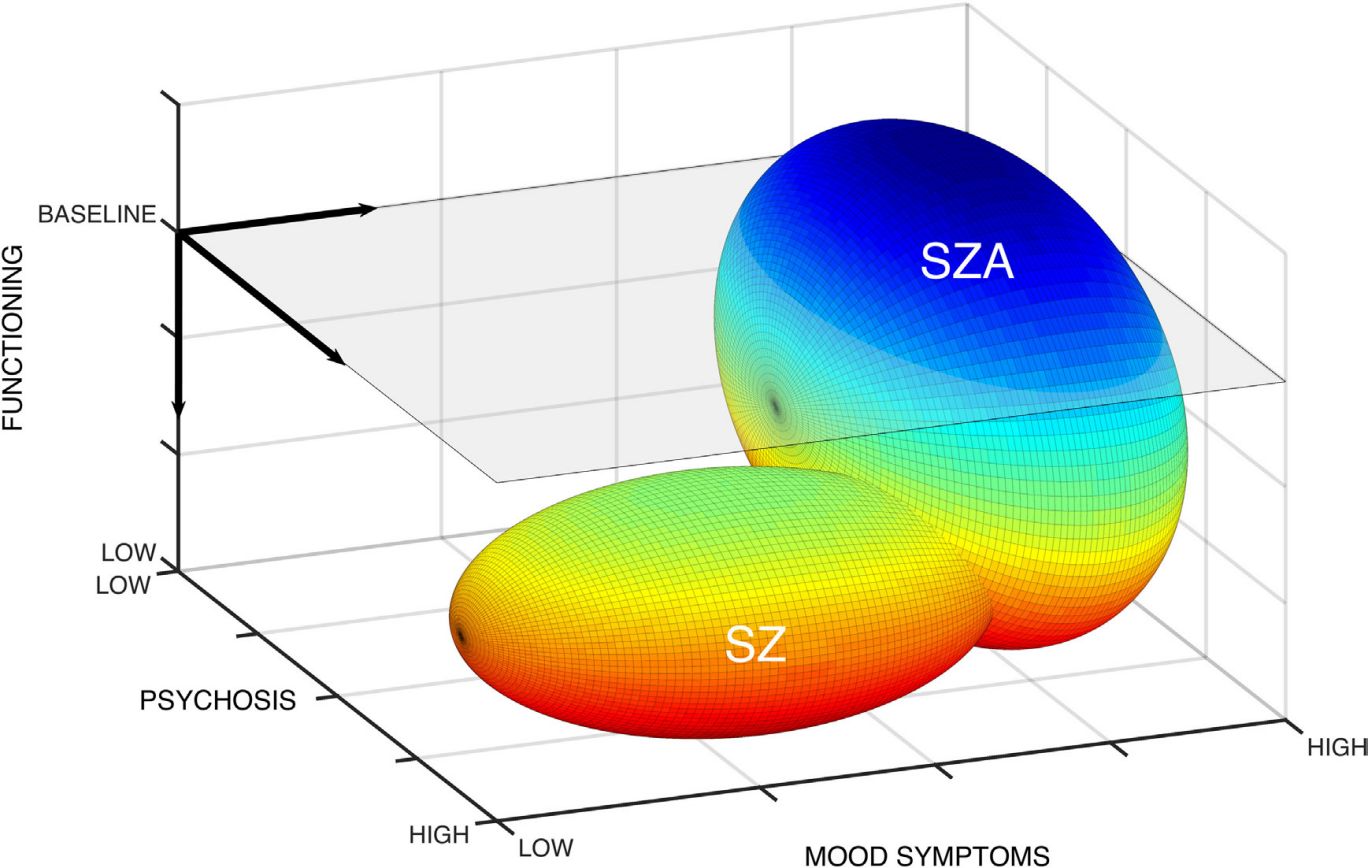
**Domains of psychopathology for schizophrenia and schizoaffective disorder**. The symptom space for schizophrenia and schizoaffective disorder is defined by three domains: psychosis, mood symptoms, and functioning. Gray plane indicates baseline functional status. Heat map with hotter colors indicates worsening functional impairments. Ellipsoids demonstrate that schizophrenia occupies a constrained symptom space low on functioning, high on psychosis, and lower mood symptoms. By contrast, schizoaffective disorder is less constrained with potential for greater heterogeneity in functional status and degree of psychosis, though high on mood symptoms.

Schizophrenia and schizoaffective disorder are defined by three domains of psychopathology: psychosis, mood symptoms, and functional impairment. Figure 1 plots the space defined by these three domains. We display the schizophrenia population as an ellipsoid constrained by two diagnostic criteria: decrease of function below the level achieved prior to illness onset (criterion B) and disturbances lasting for at least 6 months (criterion C). Mood symptoms may be present but are not major. Criteria B and C were added as gatekeepers during the DSM-III revision process, with the goal to exclude less severe cases and to increase the reliability and validity of schizophrenia (3).

By contrast, the schizoaffective disorder population occupies a considerably larger space, including patients with no functional impairment and with lower levels of psychosis. Within these differently sized spaces, we diagnose schizophrenia three times more often than schizoaffective disorder (4). The result is a homogeneous schizophrenia population and a heterogeneous schizoaffective disorder population.

In an attempt to reduce diagnostic heterogeneity, Williams and McGlashan employed a stringent definition of schizoaffective disorder: patients met DSM-III diagnostic criteria for both schizophrenia and an affective disorder (5). Their schizoaffective disorder cohort did not differ from the schizophrenia cohort on a number of demographic, clinical, and outcome variables. They recommended that diagnostic criteria should include a specification of whether schizoaffective disorder patients also meet full schizophrenia criteria, in order to reduce heterogeneity and minimize conflicting findings.

Here, we build on the study by Williams and McGlashan by comparing patients who met DSM-IV-TR criteria of schizoaffective disorder and schizophrenia, respectively. While the DSM-IV-TR diagnoses schizophrenia and schizoaffective disorder share criterion A (delusions, hallucinations, disorganized speech, disorganized or catatonic behavior, and negative symptoms), the schizophrenia criterion B (marked functional impairment) and criterion C (continuous signs for at least 6 months) are permissible, but not required for a diagnosis of schizoaffective disorder (Table 1). This results in considerable heterogeneity, since the schizoaffective disorder population includes patients who do or do not meet schizophrenia criteria B and C.

**Table 1:** **DSM-IV-TR diagnostic criteria comparison**.

Criterion		DSM-IV-TR schizophrenia	DSM-IV-TR schizoaffective disorder	Current schizoaffective disorder sample
A	2 or more of 5 symptoms	Yes	Yes	Yes
B	Marked functional impairment	Yes	?	Yes
C	Continuous signs for at least 6 months	Yes	?	Yes
D	Mood symptoms are brief	Yes	No	No
E	Not due to substance abuse/medical condition	Yes	Yes	Yes

*? represents the diagnostic uncertainty in the DSM-IV-TR schizoaffective disorder criteria regarding functional impairment and duration of illness*.

In this study, we compared a group of schizoaffective disorder patients, who met DSM-IV-TR diagnostic criteria for schizoaffective disorder and also met DSM criteria B and C for schizophrenia, with a group of schizophrenia patients. The two groups were comparable in terms of sex, age, race, and duration of illness. We hypothesized that the groups would not differ with regard to antecedent risk factors, clinical features, and functional outcome.

## Subjects and Methods

### Subjects

The sample for this study was taken from an ongoing data repository at Vanderbilt University Medical Center, the Psychiatric Genotype/Phenotype Project. Subjects were recruited from inpatient units (61% of schizoaffective disorder sample, 54% of schizophrenia sample) and the outpatient clinic (23% of schizoaffective disorder sample, 23% of schizophrenia sample) of an academic medical center, and a local community mental health clinic with many Medicaid/Medicare patients (16% of schizoaffective disorder sample, 23% of schizophrenia sample). Subjects with a schizophrenia or schizoaffective disorder diagnosis were identified by medical record review and approached for participation in the repository. All patients enrolled were free of significant head injury, major medical or neurological illness, and active substance abuse or dependence. All study procedures were approved by the Vanderbilt University Institutional Review Board (Nashville, TN, USA), and written consent forms accompanied by comprehensive explanations of all study procedures were provided. Subjects were given monetary compensation for their participation.

The sample for the present study included all subjects who met diagnostic criteria for schizophrenia (*N* = 114) or schizoaffective disorder (*N* = 62) in the repository. Details concerning diagnostic criteria and review are outlined below.

### Clinical Assessment

Study subjects were interviewed by Bachelors/Masters educated research assistants, who had completed intensive training, lasting a minimum of 3 months, by clinicians with expertise in conducting structured clinical interviews. Additional information from inpatient and outpatient medical records was obtained when available. All diagnoses were then reviewed and confirmed during consensus diagnostic meetings with an expert psychiatrist (Stephan Heckers).

All subjects were interviewed with the Structured Clinical Interview for *Diagnostic and Statistical Manual of Mental Disorders, Fourth Edition, Text Revision* (SCID-IV-TR) (6).

The reliability of the DSM-IV-TR schizoaffective disorder diagnosis is low, largely due to criterion C (mood episodes must be present for a “substantial” portion of the duration of psychosis) (7), which has a test–retest reliability kappa of 0.46 (8). In order to increase reliability of our assessment, we added the schizoaffective disorder module of the Diagnostic Interview for Genetic Studies (DIGS) (9). The DIGS module explicitly defines “substantial” as at least 30% mood episode overlap for the duration of psychosis. The majority of our schizoaffective disorder cohort (42 out of 62 = 68%) also met DSM-5 criteria for schizoaffective disorder, which required that symptoms meeting criteria for a major mood episode were present for the majority (>50%) of the duration of the psychotic illness.

All of our schizophrenia and schizoaffective disorder patients met DSM-IV-TR criteria A–C for schizophrenia. All patients met not only the DSM-IV-TR criterion A but also the DSM-5 criterion A for schizophrenia, i.e., they presented with at least two criteria A, at least one being delusions, hallucinations, or disorganized speech. Criterion B (social/occupational dysfunction) was defined as having functioning “markedly below the level achieved prior to the onset” in at least one of the following areas: (a) work, (b) personal relations, or (c) self-care. We considered an additional fourth area, (d) receiving social security disability payments for psychiatric illness, when making these judgments (evidence of significant functional impairment is necessary in order to receive social security disability payments).

Distress or impairment is also a diagnostic criterion for a major depressive episode (DSM-IV-TR and DSM-5 criterion B) or manic episode (DSM-IV-TR and DSM-5 criterion C). However, the functional impairment in schizophrenia is more severe and of longer duration, leading to a level of functioning below the level achieved prior to the onset. All schizoaffective disorder patients in this study had significant impairment in at least one of the four impairment areas, and over half had impairment across all areas (Table 2), making this a sample with a high degree of functional impairment. While the DSM-IV-TR criterion C for schizophrenia (continuous signs for at least 6 months) includes the prodromal, active, and residual phases of psychosis, all subjects in this study experienced at least 6 months of active psychosis in addition to any prodromal or residual phases.

**Table 2:** **Impairment**.

Impairment area	One impairment (# of subjects)	Two impairments (# of subjects)	Three impairments (# of subjects)	Four impairments (# of subjects)	% impaired per area
Work	3	2	22	35	100
Personal relations	0	1	20	35	90
Disability	0	1	13	35	79
Self-care	0	0	11	35	74

For further characterization of the patient sample and to assess dimensions of current psychopathology, we complemented the SCID interview with the Global Assessment of Functioning (GAF) scale, the 17-item Hamilton Depression (HAM-D) rating scale, the Young Mania Rating Scale (YMRS), and the Positive and Negative Syndrome Scale (PANSS). We used the Wechsler Test of Adult Reading (WTAR) to assess premorbid IQ as an antecedent factor and the Screen for Cognitive Impairment in Psychiatry (SCIP) for the assessment of current cognitive function. We assessed functional outcome by collecting information about marital status, employment history, disability status, number of hospitalizations, and number of arrests during the SCID interview.

### Statistical Analysis

We tested for group differences between the schizophrenia and schizoaffective disorder patients. Kolmogorov–Smirnov, skewness, and Kurtosis tests for normality revealed that a number of our continuous variables were not normally distributed. We used non-parametric Mann–Whitney *U* tests to assess all continuous variables (age; duration of illness; CPZ equivalent and medication history; years of education; HAM-D, YMRS, PANSS, GAF, WTAR, and SCIP scores; medication history, arrests, psychiatric hospitalizations, hospitalizations to prevent suicide, and suicide attempts). Pearson chi-square analyses were used to assess categorical variables (sex, race, marital status, employment, disability, substance use disorders, and anxiety disorders).

## Results

### Sample Characteristics

Subjects were comparable with regard to sex, age, race, and duration of illness (Table 3). The groups had a similar history of treatment with antipsychotic and antidepressant drugs and were treated with a similar dosage of antipsychotic drugs at the time of the interview (Table 3). The schizoaffective disorder patients had been treated with a significantly greater number of mood stabilizers (*U* = 2,403.50, *p* < 0.01) (Table 3).

**Table 3:** **Sample characteristics**.

	Schizoaffective disorder (*N* = 62)	*N*	Schizophrenia (*N* = 114)	*N*	Significance	Different?
**Sex**						
% male	55	62	63	114	0.281	=
% female	45		37			
**Age**						
Years	37.13 ± 11.95	62	36.59 ± 12.02	114	0.801	=
**Race**						
% White	68	62	54	114	0.224	=
% Black	27		40			
% others	5		6			
**Duration of illness**						
Years	15.34 ± 10.12	48	16.58 ± 12.26	93	0.659	=
**Medication**						
Current antipsychotic dose (CPZ equivalent)	589.14 ± 551.80	54	548.54 ± 303.54	107	0.634	=
Historical # of antipsychotics	4.80 ± 2.62	60	4.04 ± 2.30	112	0.061	=
Historical # of mood stabilizers	1.88 ± 1.44	60	1.22 ± 1.16	112	0.001*	SZA > SZ*
Historical # of antidepressants	2.80 ± 2.43	60	2.04 ± 2.10	112	0.039	=

*Significance survived the Bonferroni correction for multiple comparisons (*p* < 0.008).

### Antecedent Risk Factors

The schizoaffective disorder group did not differ from the schizophrenia group on two measures of premorbid function associated with the risk for psychosis: premorbid intellectual function, as assessed with WTAR (*U* = 3,288.00, *p* > 0.05), and years of education (*U* = 3,174.00, *p* > 0.05) (Table 4).

**Table 4:** **Validators**.

	Schizoaffective disorder (*N* = 62)	*N*	Schizophrenia (*N* = 114)	*N*	Significance	Different?
**Antecedent risk factors**						
IQ: Wechsler Test of Adult Reading	95.89 ± 16.82	61	94.48 ± 17.39	114	0.555	=
Years of education completed	13.25 ± 2.61	62	12.67 ± 2.26	114	0.257	=
**Clinical features**						
Global Assessment of Functioning	42.89 ± 12.99	56	42.48 ± 13.67	106	0.929	=
Cognition: Screen for Cognitive Impairment in Psychiatry total z-score	−1.57 ± 1.05	57	−1.63 ± 1.03	110	0.807	=
Hamilton Depression score	11.95 ± 6.73	61	8.50 ± 6.58	113	0.001*	SZA > SZ*
Young Mania Rating Scale score	6.65 ± 7.12	51	4.82 ± 4.50	98	0.302	=
Positive and Negative Syndrome Scale (PANSS) positive score	19.57 ± 6.68	61	20.45 ± 7.05	110	0.369	=
PANSS negative score	13.62 ± 4.85	61	16.61 ± 7.50	110	0.028	=
PANSS general score	33.57 ± 7.79	61	33.17 ± 8.28	110	0.722	=
PANSS total score	66.77 ± 15.02	61	70.24 ± 17.22	110	0.169	=
% comorbid substance abuse	66	62	61	114	0.463	=
% comorbid anxiety disorder	48	62	19	114	<0.001*	SZA > SZ*
% PTSD	21	62	4	114	<0.001*	SZA > SZ*
# of suicide attempts	4.58 ± 10.98	62	1.08 ± 2.43	114	<0.001*	SZA > SZ*
# of hospitalizations to prevent suicide	3.05 ± 4.91	56	1.57 ± 4.24	110	0.001*	SZA > SZ*
**Functional outcome**						
% impaired marital status	89	62	92	114	0.454	=
% employed	18	62	18	114	0.974	=
% disability	79	62	83	114	0.480	=
# of hospitalizations	10.18 ± 11.08	57	9.69 ± 21.53	110	0.142	=
# of arrests	3.63 ± 12.88	62	3.07 ± 7.16	113	0.774	=

*Significance survived the Bonferroni correction for multiple comparisons (*p* < 0.003).

### Clinical Features

Both groups displayed moderate degrees of psychosis (PANSS scores) and few signs of mania (YMRS scores). The schizoaffective disorder patients were more depressed than the schizophrenia patients (HAM-D scores of 12.0 versus 8.5; *U* = 2,276.00, *p* < 0.01). Patients with schizoaffective disorder also reported more suicide attempts (*U* = 2,302.00, *p* < 0.01) and more hospitalizations to prevent suicide (*U* = 2,183.50, *p* < 0.01).

Both groups showed similar rates of substance use disorders comorbidity [χ^2^(1, *N* = 176) = 0.54, *p* > 0.05], but the schizoaffective disorder group showed a higher rate of anxiety disorders [χ^2^(1, *N* = 176) = 16.32, *p* < 0.01], particularly PTSD [χ^2^(1, *N* = 176) = 14.03, *p* < 0.01].

The GAF scores did not differ between the two groups. The two groups also did not differ in their performance on the SCIP, which provides a measure of cognitive function at the time of the interview.

### Functional Outcome Data

There was no difference between the two groups on a variety of measures of functional or clinical outcome, including history of marital status [χ^2^(1, *N* = 176) = 0.56, *p* > 0.05], number of arrests (*U* = 3,414.00, *p* > 0.05), percentage of patients employed [χ^2^(1, *N* = 176) = 0.001, *p* > 0.05], percentage qualifying for social security disability [χ^2^(1, *N* = 176) = 0.50, *p* > 0.05], or number of hospitalizations (*U* = 2,701.00, *p* > 0.05).

## Discussion

Previous comparisons of schizoaffective disorder and schizophrenia have been hampered by significant heterogeneity of the schizoaffective disorder samples. Here, we present results from a comparison of patients who met DSM criteria A–C for schizophrenia but differed with respect to meeting DSM criteria for schizoaffective disorder. When defined with this stringency, schizoaffective disorder and schizophrenia show similarities in antecedent risk factors, clinical features, and functional outcome. However, the schizoaffective disorder patients were more depressed, reported greater suicidality, and showed increased rates of anxiety disorders, especially PTSD. Trauma shapes the phenotype of psychosis (10), but whether this affects schizoaffective disorder patients more than schizophrenia patients warrants further examination.

These results differ from the conventional view that schizoaffective disorder is a more benign clinical condition than schizophrenia (11). But they are supportive of recent reports that inconsistent clinical characterization of schizoaffective disorder, including the use of multiple diagnostic criteria, has led to poor consensus across studies (1, 2). To our knowledge, only one previous study restricted the schizoaffective disorder sample to those patients who also met full schizophrenia criteria, and the authors reported a comparable clinical picture for their two study cohorts (5). Employing a similarly stringent set of criteria, our results indicate comparable clinical and functional outcomes for schizoaffective disorder and schizophrenia.

Schizoaffective disorder has been defined in the context of a categorical diagnostic system, but there is compelling reason to redefine the disorder in the context of a dimensional system (12). The two patient groups in our study were comparable on the majority of measures but showed higher rates of anxiety disorders and suicidality. A mixed categorical (schizophrenia criteria A–C) and dimensional (mood and anxiety symptoms) definition of schizoaffective disorder would be a reasonable alternative to the current DSM-5 definition of schizoaffective disorder. Psychotic patients can then be assessed for affective symptoms without imposing a categorical distinction between schizophrenia or schizoaffective disorder. Levitt and Tsuang suggested subtyping of schizoaffective disorder patients based on the polarity of mood symptoms (depressive and manic subtypes) (13), as is present in the DSM-IV-TR and ICD-10 classifications (14). However, McGlashan and Williams suggested that attention should be paid instead to how “schizophrenic” the schizoaffective disorder clinical picture is, and that this will be the strongest predictor of outcome (15). This suggestion is similar to the “Mainly Schizophrenic” subtype in the RDC classification, which requires the presence of psychosis for at least one week outside of a mood episode (16), and the optional specification of psychosis outside of mood symptoms in the ICD-10 classification.

Schizoaffective disorder is a common clinical diagnosis (17), despite numerous concerns regarding validity, reliability, and clinical utility (7, 18–21). As diagnostic criteria utilized in schizoaffective disorder research greatly vary, the findings of the present study emphasize the importance of using well-defined diagnostic criteria when examining schizoaffective disorder. Defining schizoaffective disorder with additional criteria for duration of psychotic episodes and functional impairment will improve the reproducibility of schizoaffective disorder research. While the DSM-5 criterion C for schizoaffective disorder has become more stringent, it will decrease prevalence rates, but not necessarily the diagnostic heterogeneity of schizoaffective disorder reported here.

We were able to examine only one subgroup of schizoaffective disorder patients, which is a significant limitation of this study. When reviewing our cohort of chronic psychosis patients eligible for inclusion in the study, only 1 of 177 met schizoaffective disorder criteria but not schizophrenia criteria B and C. This might indicate a greater prevalence of the schizoaffective disorder subpopulation examined here; however, we need to consider an ascertainment bias since our patient sample was primarily recruited from psychiatric inpatient units. It is an essential next step to compare schizoaffective disorder patients, who do not meet schizophrenia B and C criteria, with the two groups considered here and with a group of psychotic bipolar disorder patients. Another limitation of our study is the narrow set of clinical and functional outcome data. Future studies should explore the antecedent, concurrent, and predictive validators of schizoaffective disorder more thoroughly.

Schizoaffective disorder has been considered a better outcome diagnosis when compared with schizophrenia, but the literature has been inconclusive. The present study supports the need for a more detailed clinical characterization of schizoaffective disorder patients for treatment and endophenotype studies, thus reducing heterogeneity and increasing reliability and validity.

## Ethics Statement

This study was carried out in accordance with the recommendations of the Vanderbilt University Institutional Review Board with written informed consent from all subjects. All subjects gave written informed consent in accordance with the Declaration of Helsinki. The protocol was approved by the Vanderbilt University Institutional Review Board.

## Author Contributions

KS, KA, and SH conceptualized the study. KS setup and performed all data analyses; wrote the original draft of and prepared the manuscript. SH, MS, and KA edited the manuscript.

## Conflict of Interest Statement

The authors declare that the research was conducted in the absence of any commercial or financial relationships that could be construed as a potential conflict of interest. The reviewer R-DS and handling Editor declared their shared affiliation, and the handling Editor states that the process nevertheless met the standards of a fair and objective review.
